# Mobile phone text-message–based drinking brief interventions for young adults discharged from the emergency department

**DOI:** 10.1186/1940-0640-7-S1-A50

**Published:** 2012-10-09

**Authors:** Brian Suffoletto

**Affiliations:** 1 Department of Emergency Medicine, University of Pittsburgh Medical Center, Pittsburgh, PA, USA

## 

Brief intervention (BI) has the potential to reduce heavy drinking in young adults that present to the emergency department (ED) but require time and resources that are rarely available. Text-messaging (TM) may provide an effective way to collect drinking data from young adults after ED discharge as well as to provide ongoing support for behavior change. Young adults in three urban EDs identified as hazardous drinkers based on Alcohol Use Disorders Identification Test-Consumption (AUDIT-C) score were randomly assigned to weekly TM feedback with goal setting (intervention group), weekly TM drinking assessments without feedback (assessment group), or a control group. Participants in the intervention group who reported drinking ≥5 drinks during any 24-hour period (≥4 drinks for women) were asked whether they would set a goal to reduce their drinking the following week. We assessed the interaction between TM and goal-setting as well as heavy drinking days (HDD), and drinks per drinking day (DPDD) using the timeline follow-back procedure at baseline and three months. Forty-five young adults (age 18-24 years, 54% female) met inclusion criteria and were randomized to one of the three study conditions. Of these, six (13%; 95% confidence interval [CI], 5-27) did not complete the three-month web-based follow-up. Eighty-eight percent (95% CI, 84-91%) of weekly TM drinking assessments were answered, with 77% (95% CI, 58-90) of participants responding on all 12 weeks. Agreeing to set a goal was associated with a repeat HDD 36% of the time (95% CI, 17-55) compared with 63% of the time (95% CI, 44-81) when participants were not willing to set a goal. At three months, participants exposed to the TM intervention had 3.4 fewer past-month HDD (standard deviation [SD], 5.4) and 2.1 fewer DPDD (SD, 1.5) compared with baseline. Interventions delivered via text message have the potential to reduce heavy drinking among young adults but larger studies are needed to establish efficacy.

